# A Review of Fluorescent Carbon Dots, Their Synthesis, Physical and Chemical Characteristics, and Applications

**DOI:** 10.3390/nano11061448

**Published:** 2021-05-30

**Authors:** Mychele Jorns, Dimitri Pappas

**Affiliations:** Department of Chemistry and Biochemistry, Texas Tech University, Lubbock, TX 79409, USA; mychele.jorns@ttu.edu

**Keywords:** carbon dots, carbon quantum dots, nanoparticles, fluorescence, bioimaging, sensing, super-resolution

## Abstract

Carbon dots (CDs) are a particularly useful type of fluorescent nanoparticle that demonstrate biocompatibility, resistance to photobleaching, as well as diversity in composition and characteristics amongst the different types available. There are two main morphologies of CDs: Disk-shaped with 1–3 stacked sheets of aromatic carbon rings and quasi-spherical with a core-shell arrangement having crystalline and amorphous properties. They can be synthesized from various potentially environmentally friendly methods including hydrothermal carbonization, microwaving, pyrolysis or combustion, and are then purified via one or more methods. CDs can have either excitation wavelength-dependent or -independent emission with each having their own benefits in microscopic fluorescent imaging. Some CDs have an affinity for a particular cell type, organelle or chemical. This property allows the CDs to be used as sensors in a biological environment and can even provide quantitative information if the quenching or intensity of their fluorescence is dependent on the concentration of the analyte. In addition to fluorescent imaging, CDs can also be used for other applications including drug delivery, quality control, photodynamic therapy, and photocatalysis.

## 1. Introduction

In fluorescent imaging, a probe or label must be used that will specifically target the desired cell, organelle or molecule of interest. The lack of native fluorophores for most analytical measurements has led to the development of a myriad of luminescent reporters. It is most common to use fluorescent dyes or proteins for this purpose. However, they are not the only option available nor necessarily the most ideal option. Nanoparticles have proven to be quite useful for fluorescence imaging, including below the diffraction limit via super-resolution microscopy, and consist of a variety of compositions, shapes, and sizes that can be engineered to meet experimental demand. Silica nanoparticles (typically doped with fluorescent dyes), polymer dots, quantum dots, carbon dots (CDs), and the numerous types of other carbonaceous materials are all common types of nanoparticles used in fluorescent and luminescent imaging [1]. CDs, a recently developed class of carbon-based nanoparticles, are the focus of this review.

CDs offer several crucial benefits as probes for fluorescent imaging of biological samples. First, the methods available for synthesizing CDs are relatively uncomplicated, sustainable, and environmentally friendly [2,3]. There can also be great variation in the chosen mode of synthesis, and therefore in the type of CD produced, between the selection of starting materials, heating method, and purification method or methods applied to the sample to isolate the CDs or version of CDs of interest [4]. Second, due to their small size and composition, CDs have been proven to be non-toxic within working conditions for microscopic fluorescent imaging [5]. CDs are able to pass through the cellular membrane and localize within the cytoplasm or even pass further into the cell and reside within its organelles.

CDs do not readily photobleach, unlike fluorescent dyes and proteins, even when they are excited by a laser for several hours [6]. Since CDs are synthesized at relatively high temperatures, compared to the normal range of temperatures in a biological specimen, they will not degrade in a solution or within samples unless this same temperature is exceeded. Additionally, CDs have the ability to endure other extreme environmental conditions such as high and low pH and high ionic strength [7,8]. CDs are often stored cryogenically for lengthy periods and have been found to retain their beneficial properties after thawing. However, they can also be stored at room temperature.

CDs also have the ability to produce excitation wavelength-dependent and/or -independent fluorescent emission [9]. For the CDs which display wavelength-dependent emission, the possibility of performing multiple images at various excitation wavelengths can reveal a complete emission profile of CDs in a sample and identify interfering fluorescence such as from autofluorescence of cells or contaminants. Excitation wavelength-independent emission allows the selection of specific wavelength(s) of emission without the need to specify the wavelength of the excitation source.

A change in intensity and/or wavelength of emission of CDs while within a sample when the excitation source is unaltered can be an indication of the presence of a particular chemical or environmental condition (temperature, pH, etc.). This behavior by CDs opens up the possibility of their use as biological and chemical sensors. However, it must be proven that the CDs are able to specify just the chemical or condition of interest and not be influenced by the additional species present.

## 2. Fabrication and Intrinsic Qualities

### 2.1. Synthesis Methods

When compared to other nanoparticles, CDs have the advantage in terms of synthesis methods since there are multiple methods available to choose from of which some are environmentally friendly. They can be synthesized through a “bottom-up” process using small organic chemicals, for instance, malic acid [10] and urea [11,12,13] or even biological materials such as biowaste [14] or animal [15] and plant products [16,17,18], that are made to chemically converge into nanoparticles [4,9,19]. Alternatively, in a “top-down” process, a large pure carbon compound, for example, carbon black [20], carbon nanotubes [21] or graphite, is fragmented into nanoparticles [9]. The bottom-up method has key advantages over the top-down method including being more environmentally friendly, less time-consuming, and allowing for easy modification of the surface state and composition of the CDs. Bottom-up methods are the more common choice in the literature for these reasons, and also make carbon dots an ideal choice over other types of nanoparticles. In bottom-up synthesis, the organic compound is dissolved in a solvent then heated to the point where the chemical undergoes dehydration and carbonization. This process can be achieved through several different techniques such as hydrothermal carbonization, microwaving, pyrolysis or combustion [4,10,19,22]. Each of these techniques has its own advantages and disadvantages in terms of time, cost, efficiency, and energy consumption, and will produce CDs of various size and composition. Some examples of proven methods for synthesizing CDs are listed in Table 1.

The hydrothermal carbonization method is very common in scientific literature and considered to be relatively simple, low-cost, and uses non-toxic starting materials for controlled CD formation such that modifications to the CD composition can be made readily [19]. This technique relies on a specifically designed reaction vessel which will withstand the necessary high temperatures for carbonization while containing all erupting vapors to increase the pressure of the reaction. With the vapors contained, the high pressures improve the efficiency of the digestion of organic materials and the sample can be heated for longer periods of time without losing volume by evaporation [23]. CDs made from this method will tend to have a high photoluminescent quantum yield (Section 3.5), but the drawbacks include non-uniformity in particle size, impurities in the product which cannot be easily removed, and possibly variation in photoluminescent behavior between CDs in the same sample [11,19].

Electromagnetic radiation in the form of microwaves can be an alternative heating method to an oven, which is used in hydrothermal carbonization. One could use a similar vessel to what is used in hydrothermal carbonization but made up of materials which are nonmetallic to also increase the efficiency of the digestion of the organic materials [24]. The main benefit of microwaving is that the strong interaction of the electromagnetic radiation and the carbon source allows for rapid and localized heating [9,10,25]. This technique is energy-saving, environmentally friendly, and considered a simpler process than most other CD synthesis techniques. However, the resulting CDs can have a large size distribution and isolation of the CDs from the solution can be difficult [19].

Pyrolysis and combustion are both versions of thermal decomposition except they differ in the atmosphere which they are conducted in since pyrolysis uses a low oxygen or oxygen-less environment while combustion requires oxygen [9,26]. These techniques will sometimes require a strong acid or base to begin the digestion of the carbon precursor, and therefore they cannot be considered environmentally friendly [9,19]. CDs made using pyrolysis will have a relatively high photoluminescent quantum yield, but the reaction time is relatively long and requires a specific reaction setup, also, the CDs are not easily separated from the product solution [19,22]. CDs made from performing combustion do not require any additional surface modification, but the photoluminescent quantum yield is lower than the other techniques [19].

**Table 1:** List of common carbon-containing precursors, solvents, synthesis methods, and purification methods used for bottom-up synthesis of carbon dots. This list is not comprehensive but instead includes some examples of chemicals/methods which are proven to produce carbon dots. These four aspects of CD synthesis could theoretically be used in different combinations to fine tune the characteristics of the resulting CDs.

Carbon Precursor(s)	Solvent(s)	Synthesis Method	Purification Methods	Reference
Citric acid	Formamide	Hydrothermal Carbonization	Filtration, Centrifugation, Vacuum Filtration	[27]
Malic acid	Water	Microwave	Dialysis, Rotary Evaporation	[10]
Urea and Citric acid	Dimethylformamide	Solvothermal Carbonization (version of hydrothermal synthesis)	Centrifugation, Freeze-drying	[28]
Citric acid	Tetraethylenepentamine	Pyrolysis	Dialysis, Vacuum Filtration	[26]
Citric acid	Water	Microwave-assisted Pyrolysis	Dialysis, Freeze-drying	[29]
Sucrose	Nitroso or Nitrobenzene	Hydrothermal Carbonization	Column Chromatography	[30]
Urea and *p*-phenylenediamine	Water	Hydrothermal Carbonization	Column Chromatography	[11]
Folic Acid	Water	Hydrothermal Carbonization	Filtration	[31]
κ-carrageenan and Folic acid	Water	Hydrothermal Carbonization	Filtration, Freeze-drying	[32]
*Allium sativum* peels (garlic)	Water	Pyrolysis	Filtration, Dialysis	[18]
*Agaricus bisporus* (mushroom)	Ethylenediamine in Water	Hydrothermal Carbonization	Centrifugation, Filtration, Dialysis	[7]
Milk	Water	Hydrothermal Carbonization	Filtration	[15]

### 2.2. Purification Methods

After the CDs have been formed, it is ideal to then purify the sample and isolate the CDs from the rest of the solution. Additionally, there may be the need to further separate the resulting CDs by size or composition to select for CDs with a particular wavelength(s) of emission. There are several methods to choose from for the initial separation of CDs from the solution including filtration, centrifugation, dialysis, and column chromatography, which will each yield various results. It is very common to include multiple techniques for purifying the sample in order to achieve the selection of a particular subgroup in the sample by the size and/or type of CDs. Nearly every procedure will include a form of filtration and centrifugation of the raw sample since these methods will efficiently perform the initial separation of the nanoparticles from larger species in the rest of the solution. Additionally, the materials for performing filtration are inexpensive and centrifuges are a very common instrument in the lab. In most experiments, after centrifugation, the desired nano-sized product was found in the supernatant while the precipitant, believed to contain larger particles and aggregates that were not captured by the filter, are then discarded [14,33]. Dialysis is another common purification method [34,35,36], however, the experimenter must choose a dialysis membrane with the appropriate pore size to avoid product loss. Usually, dialysis will be used to remove the unreacted product and any particles which are smaller than the desired CDs, although multiple membranes of different molecular weight cutoff values can be used simultaneously to select a small range in size of CDs.

Although the methods that have been mentioned provide a way to remove unwanted material from the sample, they do not isolate the solid particles from the solution, unless the experimenter is able to isolate the nanoparticles of interest in the precipitant rather than the supernatant when centrifuging [27], therefore an additional step must be taken to remove as much of the solvent as possible such as vacuum filtration or rotary evaporation. It is crucial that, if the solvent is to be removed with heat, that the temperature is carefully monitored so that the particles are not heated to a point which would induce aggregation or excess carbonization to compromise the CDs.

An additional method of purification that can be utilized is column chromatography. With this method, the resulting fractions which have been separated by size, polarity or other means allows the experimenter to observe the range in characteristics of CDs produced from the reaction. In one example, Zhi et al. used column chromatography with diluted methanol as the mobile phase and C_18_ reversed-phased silica gel as the stationary phase to separate by decreasing polarity [10]. The emissions from each fraction under UV light (wv = 365 nm) revealed that a slight red-shift occurred with decreasing polarity and so through this purification method they had the ability to select for whichever particles had emissions in their desired section of the light spectrum. Different techniques for column chromatography can also be used in a similar way to separate the types of CDs present in a sample, for example, high-performance liquid chromatography. With this method, although the efficiency is greatly increased and the required time for completing the process is greatly reduced, only a small portion of the sample can be analyzed at a time, and therefore the overall yield is limited.

### 2.3. Morphology and Composition

**Figure 1:**
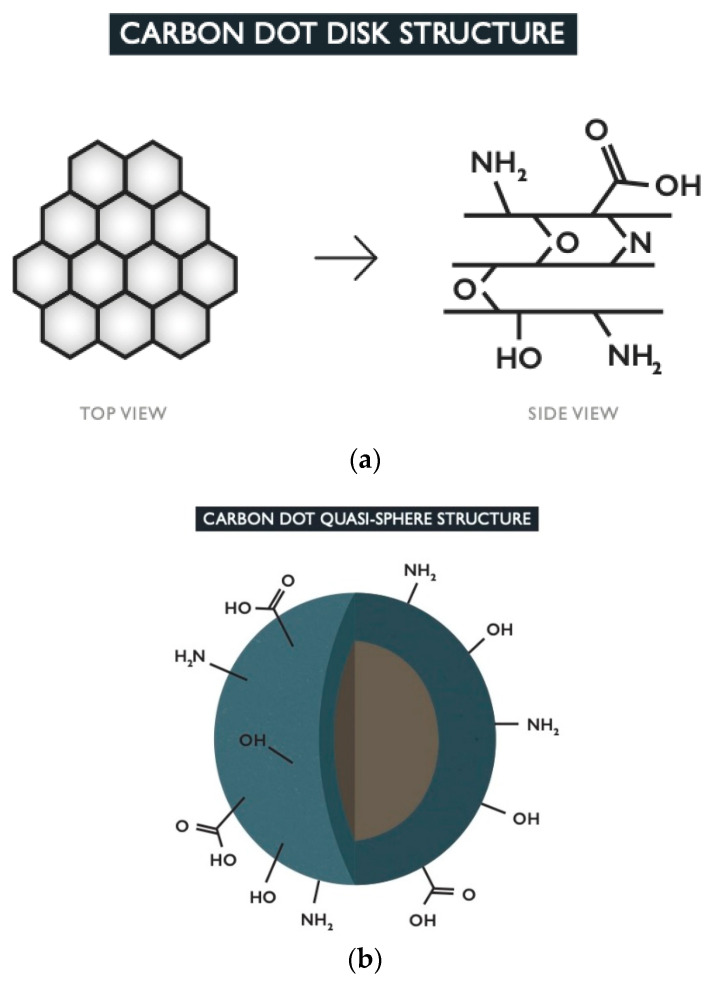
The top image (**a**) depicts the top and side views of the disk-shaped morphology. These types of CDs consist of graphene-like sheets which have a structure composed of aromatic carbon rings linked together in a honeycomb schematic. The sheets can be held together by bonds formed with dopants or by weak intermolecular forces. Functional groups formed from the dopants are present on the surface of the CD. The bottom image (**b**) shows the typical quasi-spherical carbon dot structure with core-shell schematic. The core (brown) contains the crystalline structure of carbon rings while the amorphous sp^3^-hybridized carbon matrix is found in the shell (blue). Any dopants present will form functional groups which preside on the outer surface of the CD.

The term carbon dot can be used to encompass two main morphologies [37]. The first is disk-shaped with an average of 1–3 layers of 2D graphene-like sheets with surface groups [38,39] (Figure 1). The second and more common morphology is a more intricate structure of quasi-spherical polyaromatic crystalline and/or amorphous network of carbon with various tunable surface groups [4] (Figure 1). Both morphologies will consist of mostly carbon atoms, due to organic or pure carbon precursors, with the addition of oxygen, nitrogen, and potentially additional dopants, depending on the elements present in the starting materials, as well as the reaction conditions such as time and temperature [9,32,34]. In one study, researchers studied the effect of changing the reaction atmosphere and found that each type of gas yielded CDs of different properties, with oxygen producing the highest degree of aromatization [25].

The actual structure of the various versions of spherical CDs can differ in complexity depending on which elements are incorporated into the carbon network and surface state. Moreover, the parameters of the particles’ formation itself will be an additional factor which is determined directly by how it is heated. The chosen synthesis method (microwave, hydrothermal carbonization, etc.), and in particular the temperature and duration will influence the level of carbonization [40]. CDs that exhibit both crystalline and amorphous structures will typically do so in a core-shell schematic with the highly ordered, sp^2^-hybridized aromatic carbon structures as its core and a more disordered sp^3^-hybridized carbon matrix as its shell [27,41,42,43]. These quasi-spherical structures could alternatively consist of a more simplistic schematic of just the crystalline core without the outer shell. The outermost region of quasi-spherical CDs will display any functional groups retained from the starting material or formed from O, N, and other dopants present, with common groups including carboxyl, hydroxyl, and amino groups [9]. Additionally, N can be located within the core and/or shell matrix in the form of graphitic, pyridinic or pyrrolic N as has been reported in several sources [32,34,40,44]. By definition, their overall size should be less than 10 nm in diameter [9,45]. Their small size makes them ideal for imaging of biological samples as this contributes to their excellent biocompatibility by allowing limited interference in the cell’s biological processes [46]. CDs will either be taken into the cell by passive diffusion or by active endocytosis, depending on the composition of the CD, the environmental conditions (particularly temperature), and the type of cell [27].

There are instances in the literature where CDs, that are not derived from graphene, are referred to as graphene quantum dots, typically of the disk-shaped morphology, due to the formation of graphene-like carbon ring structures within the particle’s composition. As a result, there is the potential for some confusion since graphene technically consists only of carbon and hydrogen while CDs usually contain more than these two elements. CDs may also be termed as carbon-based quantum dots or carbon quantum dots, even though the term quantum dots (QDs) refers to semiconductor quantum dots and there are many crucial differences between these two types of nanoparticle. In particular, QDs have a broader range in size (<5 nm to 10 s of nanometers) [47] than CDs with the larger sized QDs being unfavored for penetrating a cell membrane. QDs are derived from starting materials containing heavy metals and are therefore less ecologically friendly and biocompatible than CDs [46]. These carbon quantum dots, and also carbon nanodots, fall under the general classification of being CDs of the quasi-spherical morphology though their actual structures can vary in complexity.

### 2.4. Resilience and Sensitivity of Carbon Dots

Since CDs synthesis occurs at higher temperatures (minimum 160 °C for bottom-up methods) [48], they typically do not degrade within the range of temperatures maintained for homeostasis in a biological environment. In several experiments, the resilience of CDs was tested over extreme pH levels, including cycling between acidic and basic states, and in each case, the particles were able to retain their structure and their ability to fluoresce under an excitation source [7,8]. In addition to their resistance to extreme environmental factors, some CDs were proven to have fluctuating PL (photoluminescence) behavior in accordance with changes to their surrounding environment, which allows them to act as sensors for the monitoring of changes in pH or temperature within and around the specified target [41]. It was theorized in these sources that the sensitivity to pH was due to hydrogen bonding between neighboring CDs or with the solvent via nitrogen- and/or oxygen-containing functional groups on their surface. This hydrogen-bonding could occur when the pH is below the pKa value of the particular functional group so it can transition into the protonated state, altering the chemical composition of the CD, hence the observed quenching or fluctuation in fluorescence. However, hydrogen-bonding may also occur under the opposite condition when the specified functional group is deprotonated. The particular groups present on the CD surface and in the media will determine whether acidic or basic conditions are necessary for eliciting hydrogen-bonding.

In addition, CDs can be durable in high ionic strength environments. Li et al. observed how the ratio of fluorescence intensities from the two emission wavelengths changed with the increasing concentration of strong ions [35]. They determined that the slight fluctuations in the ratio of the intensities under these conditions were negligible, demonstrating the durability of CDs. CDs have also been shown not to degrade when kept in a refrigerator or cryogenic storage, even after an extended time. This makes it possible to manufacture a large quantity all at one time and store the excess for later experiments to minimize the time for sample preparation later on [49].

## 3. Fluorescence Properties

### 3.1. Excitation Wavelength-Dependent and -Independent Emission

Fluorescence from CDs can range in emission from the blue region to the near-IR region and the reasons for this variation in emission has been attributed to different factors [9,33,36,40,44,50]. Most commonly, a change in wavelength of the excitation source can produce a shift in wavelength of the emission peak, which has been demonstrated by several types of CDs [8,51,52], with direct correlation between excitation and emission wavelengths being typical. However, the fluorescence of CDs has been reported as both wavelength-dependent, as described, as well as wavelength-independent where emission does not shift dramatically and directly with the excitation source [53]. Both versions have their own benefits including high tunability and versatility or on the other hand, a more typical fluorophore behavior that can be easily anticipated, with excitation wavelength-dependence and -independence, respectively. In some types of CDs, the researchers were able to control which region of the spectrum their CDs emitted by synthesis parameters such as the choice of starting materials and/or solvent, and heating methods. Ding et al. performed several experiments for the synthesis of CDs using varied solvents and found that they could tune the photoluminescence from the blue region of the light spectrum through to the near infrared region even when the excitation wavelength is unchanged [44]. They determined that the carbon cores and the surface states were being controlled by the different reactions with the various solvents, and therefore produced the steady shift in emissions as the graphitic nitrogen content and the particle size increased for the final CDs obtained. Lu et al. stated that decreasing the temperature of the reaction was the reason for an observed shift in emission from their CDs from the blue to the red region [40]. Upon analysis of their products, they found that C=O and graphitic nitrogen were most prevalent in the red-emitting CDs and least prevalent in the blue-emitting CDs. Supposedly, this was due to an increasing rate of carbonization with the increased reaction temperature, and so they attributed these O- and N-containing groups to the tuning of the PL. Still another group found a way to synthesize the full range of emission from blue to red in a one-pot reaction of selective starting products that were reacted at 160 °C for 10 h then separated by silica column chromatography [11]. They observed that each of the fractions collected contained nanoparticles that emitted at a specific wavelength which increased from 440 nm all the way to 625 nm as the fractions increased in polarity due to a change in the surface state from increasing oxidation. Therefore, it is reasonable to assume that it is a combination of several experimental factors which influence the characteristics of the complex fluorescence emission behavior from CDs by changing the particle’s features such as particle size, amount of C=C bonding in the carbon framework, nitrogen and/or oxygen doping into the carbon matrix, functional groups present in the surface state, and other aspects that have not yet been considered.

The different emission wavelengths of CDs may provide various benefits based on their intended applications. For example, blue-emitting CDs would not necessarily be ideal for imaging in vivo as the excitation source would have to come from a region of the light spectrum with a smaller wavelength, as in from the UV region, which can be harmful to live specimens. However, red-emitting CDs can circumvent this issue by providing adequate fluorescence for imaging of this kind through the use of visible light as the excitation source. Additionally, autofluorescence from the biological sample is weaker in the red region of the light spectrum, and therefore interference from the background during imaging would be limited.

### 3.2. Theoretical Origin of Fluorescent Behavior

While the origin of the distinctive photoluminescent behavior of CDs is not known at this time, there are several theories to explain it including electronic transitions inside the aromatic carbon rings, contributions from surface trap states, and the existence of multichromopheres or multifluorophores within single particles [9,41].

As it would be quite difficult to isolate the determining factor and conclusively prove or disprove any of the listed theories of the origin of CDs fluorescence, they must all be considered as plausible at this time. Moreover, there could potentially be a combination of multiple factors which contribute to certain emissions in particular regions in the light spectrum and/or the overall emission. The common thread amongst these theories is the notion that the observed characteristics of the fluorescence behavior of carbon dots can be attributed directly to their particular chemical structures. CDs will contain some amount of polyaromatic hydrocarbon molecules either in the core of quasi-spherical CDs or in the graphene-like sheets of disk-shaped CDs as previously stated. The presence of these aromatic chemical structures allows for easy energy transfer throughout the particle via conjugation. Absorption in the UV-Vis light region by CDs is attributed to higher energy π-π* optical transitions of, for example, aromatic C=C or C=N bonds and lower energy *n*-π* optical transitions of, for example, C=O bonds [3,41].

Doping by O, N, S or other elements changes the electronic structure of the nanoparticle, and therefore the band gaps between energy levels, due to the differing electronegativity from carbon and hydrogen and potential lone pairs of electrons. This change in energy levels from the basic hydrocarbon structure can shift the observed emission to longer wavelengths.

There is also the possibility that molecular fluorophores could become incorporated into the carbon structure and retain their fluorescence capabilities. If more than one kind of fluorophore with different absorbance and emission characteristics are present in the CD, this could explain the excitation-wavelength dependent emission [41]. As each fluorophore is excited by its ideal excitation wavelength, they will display their particular emissions which could overlap with each other slightly to give the illusion that the fluorescence is continuous across the range of excitation wavelengths.

### 3.3. Photostability

**Figure 2:**
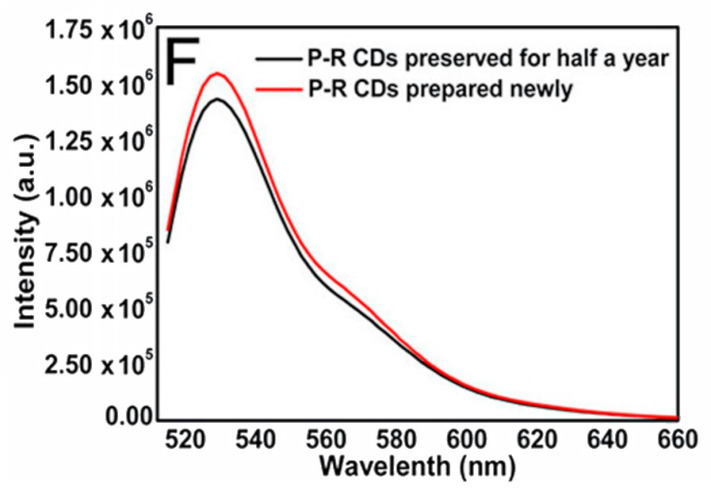
Diagram of fluorescence intensity of emissions from synthesized CDs versus wavelength of excitation source after either being stored at 4 °C for 6 months (black line) or being freshly prepared (red line). Peak intensity does diminish but not to a significant degree where CDs are no longer viable. Reprinted with permission from Tong et al. [48]; copyright 2020, American Chemical Society.

The stability of the CDs helps overcome one of the biggest problems encountered in fluorescence imaging. Photobleaching is very problematic when using fluorescent dyes, since it limits the ability to obtain usable images of the illuminated sample. With the increased excitation intensity, the time to photobleach becomes shorter, to the point where a strong enough light source can photobleach in less than 1 s [54]. Photobleaching can occur under natural light, therefore the experimenter must also be cautious with storing the fluorescent material and when preparing the sample. The CDs resistance to decrease in fluorescence with greater exposure time negates all of the previous limitations. Several images can therefore be collected in as much time as needed. When irradiating a sample containing CDs for an extended period of time, as in several hours at a time, the sample demonstrated excellent photostability and resistance to photobleaching [51]. As mentioned previously, the inherent resilience of CDs allows them the ability to be stored in a refrigerator or cryogenic storage for great lengths of time and still retain their structural integrity [49]. Specifically, experimenters tested how the fluorescence properties of CDs changed, if at all, after being stored for 6 months at 4 °C and it was found that the emission maintained its stability and intensity (Figure 2) [48].

### 3.4. Photoblinking

An interesting characteristic of some types of CDs is the spontaneous stochastic photoblinking that allows these nanoparticles to be used as fluorescent probes for super-resolution imaging [20]. Super-resolution can be achieved through various techniques, and with any of the localization methods it is crucial to be able to isolate a small portion of the sample at a time for imaging so as to avoid overlapping the emissions from neighboring fluorophores. Typically, to achieve this either a special type of photoswitchable fluorescent dye is required or the addition of devices such as phase masks or interference grids to modify the excitation profile so as to excite a select population of fluorophores at a time within the sample [55]. Images must be obtained of each isolated portion of the sample so that, when the images are compiled, the entire population of fluorophores within the field of view is captured and the final super-resolved image is produced. However, the special photoswitchable dyes will still eventually become photobleached similar to other fluorescent dyes. Moreover, super-resolution imaging techniques which require modification of the excitation profile can involve complex imaging processing [55]. Since some CDs naturally have the ability to transition from an “on” state to an “off” state under continuous illumination, the experimental setup and settings for the excitation source do not need to be adjusted to perform super-resolution imaging [10,20]. Therefore, the facile manufacturing and application of CDs for super-resolution imaging, as well as their resistance to photobleaching, helps overcome the current limitations in single-molecule imaging.

### 3.5. Quantum Yield

The high quantum yield (QY) from CDs aids in the localization of particles for super-resolution. With more photons being emitted per particle, the signal-to-noise ratio is increased. There are mathematical operations that can be done to calculate the center of a particle and determine its position through its emission profile, and this is more accurately done when the quantum yield is relatively high [31].

(1) $$QY_{sam}=QY_{ref}\frac{I_{sam}A_{ref}n_{sam}{}^{2}}{I_{ref}A_{sam}n_{ref}{}^{2}}\;$$

Equation (1) provides a means to calculate the relative quantum yield of a substance in relation to a reference material which has a naturally high quantum yield [31]. The terms “*sam*” and “*ref*” refer to the sample of CDs being analyzed and the reference material, respectively. Typically, quinine sulfate is used as the reference material. “*I*” refers to the emission intensity of the sample and reference at a specified excitation wavelength, “*A*” refers to the UV-Vis absorption intensity of the sample and reference material at the same specified wavelength, and “*n*” refers to the refractive index of the sample and reference.

The quantum yield of a sample can also be determined by the absolute method which does not rely on a reference material, but instead measures the actual number of photons absorbed then emitted [56].

(2) $$QY^{true}=\frac{QY^{obs}}{1-a+a\times QY^{2}}\;$$

Equation (2) is the formula used to calculate the absolute or “*true*” quantum yield (*QY^true^*) [56]. “*QY^obs^*” is the observed quantum yield that is calculated by the fluorimeter as a ratio of the integrated trendlines for light absorbed by the sample and light emitted by the sample. The variable “*a*” is the area of re-absorption or the difference in the overlapping integrated emission trendlines for the “*true*” emission and the observed emission which are each obtained by appropriate adjustments to the fluorimeter, its software, and the concentration of the sample. This re-absorption occurs when photons emitted in a scattered fashion by a sample are able to reenter the sample to some extent and become absorbed again. This results in a falsely low intensity of fluorescence measured for the material.

## 4. Applications

### 4.1. Bioimaging and Sensing

When CDs were first introduced as potential probes for fluorescent bioimaging, their relative toxicity to cells had to be evaluated to prove their viability for this application since other carbon-based nanoparticles available at that time were considered harmful to humans to some extent [5]. The composition of the surface of the CDs will determine their water solubility and ultimately influence their biocompatibility since the internal aromatic carbon structure of the CD does not directly interact with the surrounding environment. The hydrophilic functional groups such as hydroxyls, carboxyls, and amines commonly found on the surface of CDs can facilitate hydrogen-bonding with water and will stabilize the interaction between CDs and this solvent [9,43]. However, if the surface were to be modified by the addition of different functional groups or chemical species, then the relative cytotoxicity of the CDs would need to be reevaluated, especially if the new groups are hydrophobic or inorganic.

**Figure 3:**
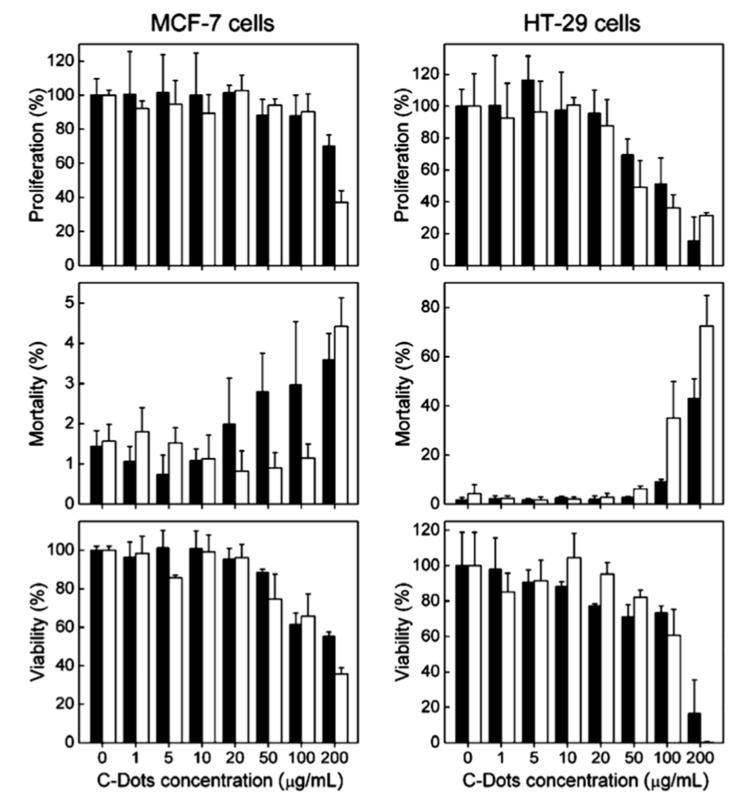
Two cell lines were incubated with various concentrations of CDs (black) and PEG_1500N_ (white). The relative toxicity of these materials is demonstrated in terms of % proliferation, % mortality, and % viability of cells. Data presented as mean ± SD (*n* = 4). Reprinted with permission from Yang et al. [5]; copyright 2009, American Chemical Society.

One group used CDs surface-passivated by the polymer PEG_1500N_ to test for cytotoxicity in terms of cell proliferation, mortality, and viability of human breast cancer cells (MCF-7) and human colorectal adenocarcinoma cells (HT-29) after incubating with the CDs [5]. The cell lines were also incubated with the polymer alone to determine if any cytotoxic effects are solely from the surface passivation agent. The resulting data from these trials is summarized in Figure 3. Ultimately, the researchers determined that, for both cell lines, the CDs had no more effect on the chosen parameters than PEG_1500N_, and therefore can be considered biocompatible for typical experiments involving cells.

**Figure 4:**
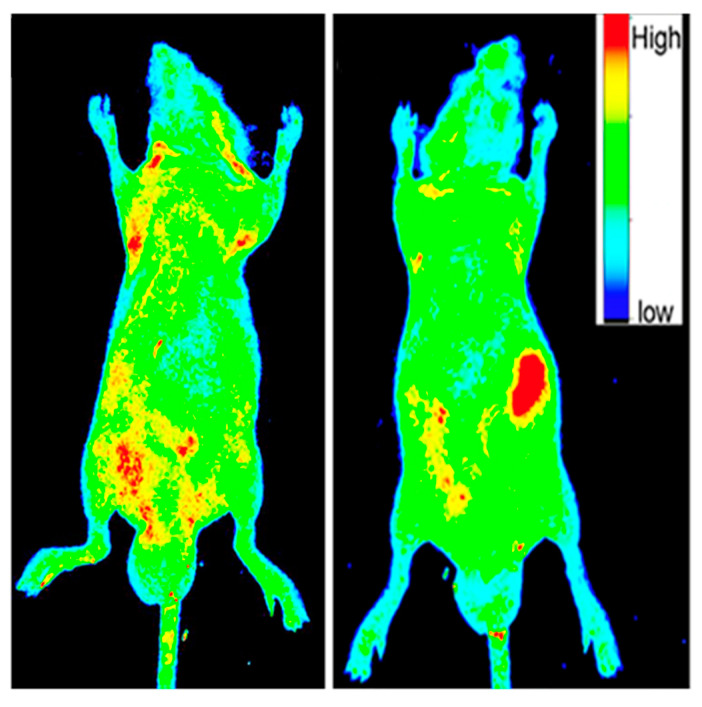
The before (**left image**) and after (**right image**) photoluminescent (PL) images under excitation light of 535 nm of a mouse which was injected with 50 µL of 1 mg/mL aqueous solution of CDs. The intensity profile of PL emissions is altered after the specimen is injected with CDs with the red regions indicating areas of high intensity fluorescent emissions. The large red region in the right image is where the CD sample was introduced into the mouse. Reprinted with permission from Ding et al. [27]; copyright 2017, American Chemical Society.

At this point in time, it is understood that since they are made from organic materials, CDs are water-soluble and biocompatible while demonstrating low cytotoxicity at working concentrations [9,32,57]. These properties open up the possibility of in vitro and in vivo fluorescent imaging. Figure 4 is taken from a paper by Ding et al. [27] which shows photoluminescent imaging of a mouse using red-emissive CDs. These images serve to demonstrate that they can function as an imaging contrast in relation to the surrounding biochemical environment due to their high intensity emissions. It should be noted that there is still extensive research needed using CDs for imaging live specimens, especially if the intent of utilizing CDs for biological imaging is to eventually move into the clinical setting [12,50].

**Figure 5:**
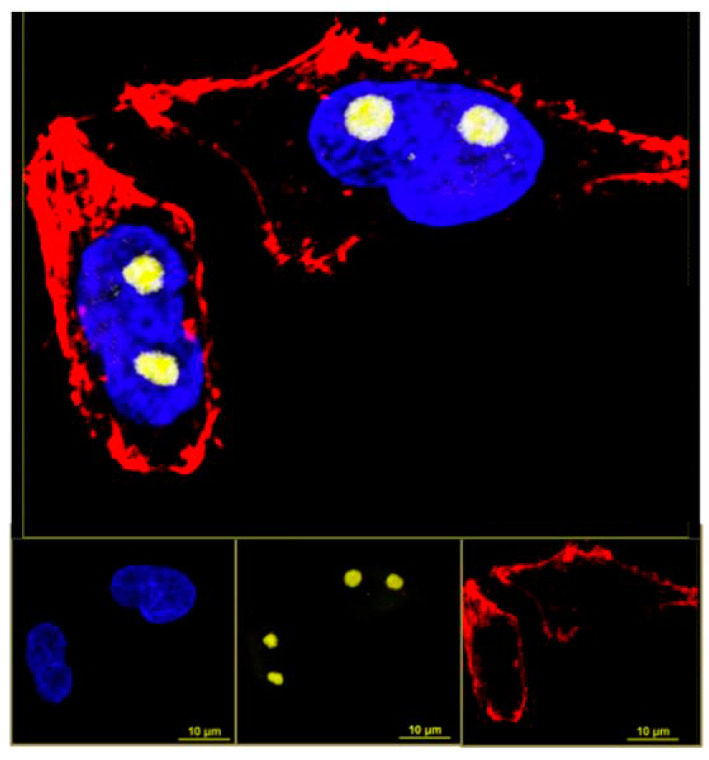
Fluorescent microscopic image of HeLa cells after staining with dyes and carbon dots. Yellow: Nucleolus labeled with carbon dots; blue: Chromatins stained with DAPI (4′,6-diamidino-2-phenylindole); red: Actin filaments stained with phalloidin conjugated with Atto647. This image demonstrates that these particular CDs target the nucleolus. Reprinted with permission from Khan et al. [53]; copyright 2018, American Chemical Society.

Each type of CD is slightly different in composition and so each will display a different affinity for cellular segments, organelles, structures or cell types and must be chosen with care when attempting to image a particular aspect of a biological sample [19]. CDs are reported in several sources as being highly specific to a particular organelle or region of a cell which will allow for high-quality imaging of the structure or structures to reveal details that were unattainable before [48,53,58]. Figure 5 shows fluorescence microscopy images taken of HeLa cells which were stained with two different dyes and CDs to illuminate different cellular structures [53]. The chosen CDs had an affinity for the nucleolus and so by using them in conjunction with certain dyes and appropriate fluorescence imaging filters, the experimenters captured a series of images which isolated the chromatins, actin filaments, and then the nucleolus to create a final stacked detailed image of the cells. Moreover, the chemical processes and physical changes occurring at that structure can be illuminated since the movement and fluorescent behavior of CDs can be monitored over an extended period of time.

The fact that CDs can display an affinity to a particular chemical opens up the possibility of utilizing CDs as sensors. However, the use of CDs as sensors requires a direct correlation between analyte concentration and fluorescent behavior (quenching, increased intensity or recovery) otherwise there would be no observable indication to the presence of the analyte. Several sources reported that their particular type of carbon dot was sensitive to a certain chemical, which can ultimately be useful in the clinical setting for diagnosing subjects that have abnormal levels of, for example, a particular hormone, protein or metal ion [35,59,60].

Since CDs have been proven to be capable of pH and temperature sensing, these two environmental factors can illuminate more information about the microcellular environment in a time-dependent, live-imaging manner at the specified location. Changes in pH and temperature in and around a cell can be the result of the natural ebb and flow of cell homeostasis, when the changes are minimal. However, when a more drastic rise or fall in these factors occurs this usually signals that a disruption to cell metabolism and viability has ensued such as the presence of a tumor [7].

### 4.2. Drug Delivery

CDs have the potential to be utilized as nano-sized drug delivery agents [43,61]. The concept is based on the principle that nanoparticles can have the ability to target specific cells, even so far as to differentiate between healthy and tumor cells, and this specificity can be utilized to deposit drugs/treatments directly at a desired site [61,62]. This enhanced permeability and retention (EPR) effect on cancer cells has been observed in different kinds of nanoparticles but is still not fully understood. By taking advantage of this process through the design of a nanoparticle-based drug delivery system, the effectiveness of the drug is enhanced overall and toxicity to the patient is reduced [61,63]. QDs have been studied for their potential to be used for these purposes since their surface can be easily conjugated to drugs and other ligands [64]. QDs may be rendered hydrophilic by the addition of hydrophilic functional groups on its surface to make them compatible with the biological system. However, there is still the drawback of QDs cytotoxic behavior which would ultimately do harm to a patient if used in the clinical setting. Other nanomaterials can also be used as drug delivery agents, such as micelles composed of lipids or amphipathic polymers which have the benefit of biocompatibility with the human body. While these nanosized vesicles can effectively protect the drug from degradation or premature release, they do not have the intrinsic targeting ability of nanoparticles [65,66]. CDs have the important benefits needed for this application to be successful including low cytotoxicity, easy surface modification, stability in a complex biochemical environment, possibly demonstrating the EPR effect, and are of such a small size that they can easily be internalized into the cell and penetrate deep tissues where the drug would not normally be able to access [29,32,61].

**Figure 6:**
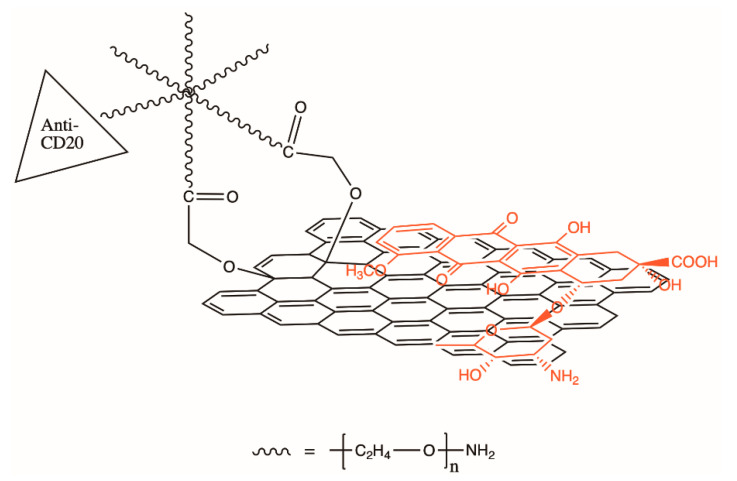
Illustration of the loading of the drug doxorubicin (DOX) via π-stacking onto nano-graphene oxide which has been conjugated with anti-CD20 antibody for cancer cell targeting. Recreated from Sun et al. [67].

A proven method for modifying a type of CD to control targeting of the desired cancer cells involves functionalizing antibodies to its surface which are specific to the type of target cell. Sun et al. used this method to deliver a cancer treatment stored between the graphene-like layers of a CD of the disk morphology to B-cell lymphoma cells (Figure 6) [67]. When the CD-drug-Ab complex entered an acidic environment (as found near tumor cells) the drug was released due to the increase in the chemical’s solubility. This concept of a three-part design with targeting agent (antibody), imaging agent (CD), and drug is also used in other drug delivery systems that may use different nanoparticles or materials [61,63].

Additionally, Li et al. used the protein transferrin, which is known to favor cancer cells due to the overexpression of the transferrin receptor on their membranes, to perform targeting of cancer cells that they postulate and can then be used in drug delivery [68]. CD nanoprobes will have to be designed to control the release of their payload only when bound to or in proximity to the target or alternatively when induced by the experimenter by external stimulation such as by ultrasound [39]. Moreover, the bond between the drug and the CD surface or its containment within the CD cannot prove too weak or too strong to be used for controlled release in a complex biological system.

### 4.3. Quality Control–Food Industry and Monitoring of Environment

In the food manufacturing industry, there are many quality checks which must occur to ensure that the product is safe for consumption and that the desired standard set by the company is being met. It is vital that any harmful or toxic substances that are present in the product be detected which creates the need for ultra-sensitive and accurate testing.

In particular, dyes are common chemicals used by food manufacturers which are necessary for creating visually appealing products. Natural dyes obtained from natural sources are generally safe but have several disadvantages in terms of performance. While synthetic dyes have the ability to endure manufacturing processes and produce color uniformity at low cost, unlike natural dyes, at times they can be harmful to humans. One group of researchers developed carbon dots whose fluorescence is quenched in the presence of the synthetic dye amaranth [13]. Amaranth is used in the manufacturing of food and drinks to induce a red color and is only nontoxic if less than 0.5 mg/kg per day is consumed. They found that the quenching of the CD’s fluorescence had a strong correlation to the concentration of amaranth and thus proved that their CDs could be used as a sensor for amaranth. Additionally, the selectivity for amaranth was tested by comparing the effect on CD fluorescence by the presence of various chemicals commonly found in real drink samples such as sugars, vitamins, salts, and amino acids. Even at concentrations 20–60 times higher than the analyte of interest, of all chemicals tested, nothing proved to significantly quench the fluorescence of the CDs in the same manner as amaranth.

The detection of foodborne pathogens is crucial throughout different branches of the food industry since contaminated products can, in some instances, prove fatal if consumed. Traditional tests for identifying harmful bacteria by microbial culture methods are limited by incubation time, inconsistent results, and can only reveal the presence of a single type of bacterium. Detection of these pathogens by nanosensors such as CDs can alternatively provide a highly sensitive, highly specific, and rapid method [69]. The CDs can be conjugated with a particular antibody that is specific to the analyte to give a qualitative and possibly quantitative analysis of the sample, if the CD does not naturally target the analyte. If the fluorescence of the CD is quenched or fluctuated in a discernable relationship with the amount of analyte present, then the fluorescence behavior will allow for a quantitative determination.

Along these same lines of quality control via sensing by CDs, certain chemicals or microbials can be detected and possibly their concentrations quantified for the purpose of monitoring of an environment such as water sources. A particular type of CD would necessarily have to be developed or modified to specify for the target of interest but, as has been demonstrated previously, CDs can naturally have this ability. In the case of detecting Sn(II) in water, Mohd Yazid et al. developed CDs which favor quenching by this metal ion even in the presence of competing metal ions such as cadmium which is commonly present in natural hard water systems [70]. As the concentration of Sn(II) increased in water or buffer solutions (pH = 5), the fluorescence intensity of the CDs decreased in a linear relationship.

Copper (II)-containing compounds and their synthesized amine-coated CDs have been used in the detection of mercury ions in tap water, lake water, and even human urine [71]. These real-world samples were each spiked with three different amounts of Hg^2+^ to create a calibration curve to ultimately determine how much mercury was present in the original specimens. While the tests with the urine sample indicated interference by autofluorescing biomolecules, the water samples demonstrated sensitive detection of Hg^2+^. The researchers even developed a paper-based sensor to allow for portable visual fluorescence detection of mercury ions by printing their CD complex in a solution form on a cellulose acetate membrane and air-drying.

### 4.4. Photodynamic Therapy (PDT)

In photodynamic therapy (PDT) for the treatment of cancer, a photosensitive molecule or specimen is utilized to generate reactive oxygen species (ROS) by transferring energy absorbed from the photons of a light source to molecular oxygen [29]. These photosensitive molecules must be deposited at or be able to target the cells or tissue which is to receive the treatment and where the light source is to be directed. These generated ROS then react with and cleave the DNA of the target cells to induce cell death. It is imperative that the ROS be localized as much as possible to the cancerous cells in order to limit the cell death of surrounding healthy tissue, therefore, illumination by the light source cannot occur until after a sufficient time for the photosensitive molecule to come in proximity to or become internalized by the target cells. For Yue et al., they were able to demonstrate through experimentation that their synthesized ruthenium-containing CDs specified for cancer cells, produced ROS when illuminated with white light and subsequently induced photocleavage of cancer cell DNA [57]. By meeting all the specified criteria, these CDs proved an ability to be applied in PDT. Other types of cancer-targeting CDs can be found in the literature which are also used in PDT [29].

### 4.5. Photocatalysis

The need for clean and sustainable energy has created a prominent area of research in the chemistry field. Photocatalysts have drawn significant attention within this field in the past decade and nanomaterials such as CDs can be used to create them. CD-based photocatalysts can be stimulated by sunlight to efficiently drive forward the chemical reactions necessary to perform the degradation of harmful organic dyes and pollutants into smaller environmentally-friendly compounds or in the photosplitting of water to produce energy by hydrogen generation.

## 5. Conclusions

Carbon dots continue to prove to be versatile in the fields of chemistry and biochemistry for sensing, detection, and labeling. Their innate biocompatibility, straightforward synthesis—relative to some organic dyes—and their amenability to single-molecule and super-resolution techniques make them attractive subjects for further study. Since CDs can be made from a wide variety of starting materials, there is still a need to investigate both their fundamental chemistry as well as all applications in chemical, biological, and material detection.

## Data Availability

The data presented in this article are available on request from the corresponding author.
